# Acid sphingomyelinase as target of *Lycium Chinense*: promising new action for cell health

**DOI:** 10.1186/s12944-016-0351-z

**Published:** 2016-10-19

**Authors:** Maria Rachele Ceccarini, Michela Codini, Samuela Cataldi, Samuele Vannini, Andrea Lazzarini, Alessandro Floridi, Massimo Moretti, Milena Villarini, Bernard Fioretti, Tommaso Beccari, Elisabetta Albi

**Affiliations:** 1 Department of Pharmaceutical Science, University of Perugia, Via Fabretti 48, 06122 Perugia, Italy; 2 Laboratory of Nuclear Lipid BioPathology, CRABiON, Perugia, Italy; 3 Department of Chemistry, Biology and Biotechnology, University of Perugia, Via Elce di Sotto 8, 06123 Perugia, Italy

**Keywords:** Antioxidant, Fatty acids, Lycium chinense, Sphingomyelin, Sphingomyelinase

## Abstract

**Background:**

Sphingomyelin plays very important roles in cell function under physiological and pathological conditions. Physical and chemical stimuli produce reactive oxygen species that stimulate acid sphingomyelinase to induce apoptosis. Antioxidant plants of the traditional Chinese Pharmacopoeia, such as *Lycium Barbarum* and *Lycium Chinense*, have become increasingly popular in Western countries. We investigated the effects of *Lycium Chinense* on acid sphingomyelinase and sphingomyelin species in relation to gene expression.

**Methods:**

We prepared *Lycium Chinense* berry extracts and evaluated their antioxidant properties. Increasing amount of extracts was used to test cytotoxic and genotoxic effect on HepG2 cells. Gene expression, protein amount and enzyme activity of acid sphingomyelinase were tested by RT-PCR, immunoblotting and enzymatic activity assay, respectively. Sphingomyelin species were analyzed by UFLC MS/MS. A panel of 96 genes involved in oxidative stress, proliferation, apoptosis and cancer was used to test the effect of LC on gene expression. GLRX2, RNF7, and PTGS1 proteins were analyzed by immunoblotting.

**Results:**

We showed that *Lycium Chinense* berries have high antioxidant properties, have an IC50value of 9.55 mg/mL, do not induce genotoxic effect and maintain high level of cell viability. The berry extracts inhibit acid sphingomyelinase activity and increase both very long fatty acid sphingomyelin species and unsaturated fatty acid sphingomyelin species. Among 96 genes, *Lycium Chinense* berries up-regulate Glutaredoxin 2 and Ring Finger Protein 7 genes and proteins, able to protect cells from apoptosis. Intrigantly, *Lycium Chinense* berries down-regulates Prostaglandin H synthase 1 gene but the protein is not expressed in HepG2 cells.

**Conclusion:**

The results identify acid sphingomyelinase as a novel target of *Lycium Chinense* berries to decrease saturated/unsaturated fatty acid sphingomyelin ratio, known to be useful for cell health. Consistent with these data, the berries regulate specifically gene expression to protect cells from apoptosis.

## Background

Sphingomyelin (SM) is an important structural/functional component of biological membranes, and one of the key-points in the synthesis and degradation of sphingolipids and glycosphingolipids. Upon stimulation, SM can be hydrolyzed to ceramide by alkaline, neutral and acid sphingomyelinases (SMase) [1, 2]. Mammalian cells contain two forms of acid SMase (aSMase), the secretory (S-aSMase) and lysosomal (L-aSMase) generated by a single gene SMPD1 [3]. L-aSMase is located within the lysosome, and S-aSMase is located extra-cellularly [4]. Recent findings have revealed the activation of aSMase in the pathophysiology of many common diseases, in apoptosis, and in different environmental and occupational stresses through the production of reactive oxygen species (ROS) and reactive nitrogen species (RNS) [5]. Free radical-mediated oxidation of SM induces activation of aSMase, which gives rise to the formation of ceramide and induces apoptosis [6].

ROS are chemical oxygen species with reactive properties, which comprise hydrogen peroxide (H_2_O_2_), the hydroxyl radical (•OH), superoxide (O^−^) and singlet oxygen (^1^O) [7]. The redox homeostasis in the cells represents the balance between cellular oxidants and antioxidants. Excessive levels of ROS induce oxidative stress that leads to various pathological states [8]. In general, most tumors exhibit higher levels of ROS than normal tissues, thus promoting tumor progression and development [9]. Marked increase in ROS can be achieved by chemotherapeutic agents, resulting in irreparable cellular damages and cancer cell death. However, some cancer cells can develop drug resistance by redox resetting [8].

Studies on antioxidant activity (AA) of fruits, vegetables and dietary supplements are increasing [10, 11]. This has led to a rapid interest in the use of plants to reduce ROS production. The antioxidant plants of the traditional Chinese medicine, such as Lycium Barbarum (LB) and *Lycium Chinense* (LC), have been increasingly popular in Western countries [12]. Investigations on the fruit have been performed mostly with LB whereas on roots and leaves have focused on LC [12]. Studies on LC fruit are really few and recent. Zhang et al. [13] demonstrated that LC fruit extract has cytoprotective effect against oxidative stress-induced hepatotoxicity. The hepatoprotective effect was supported by Ahn et al. [14]. LC fruit has been described to have neuroprotective effect on trimetyltin-induced learning and memory deficits [15], against rotenone-induced neurotoxicity [16], and in Alzheimer disease [17].

Despite the observations on chemical compounds of LC fruits and on their effect in different pathologies are expanding, no data exist about the molecular target in the recipient cell. In this study we investigated for the first time the role of LC fruit extract in aSMase modulation, and found inhibition of aSMase activity, saving of very low fatty acid (FA) SM, decrease of saturated/unsaturated FA SM, over-expression of GLRX2 and RNF7 anti-apoptotic genes and inhibition of PTGS1 cancer gene. The positive effect of LC berries in cell health was discussed.

## Results

### Antioxidant properties of *Lycium Chinense* berries and their effect in HepG2 cells

**Fig. 1:**
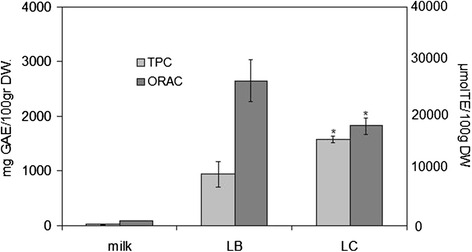
Antioxidant properties of LC berries. Total phenolic content (TPC) and antioxidant capacity (ORAC) were tested as reported in Methods. Bow milk, negative control; LB berry extract, positive control. Data are expressed as mean ± SD of three independent experiments performed in duplicate. Left ordinate, TPC; right ordinate, ORAC. (Significance, *P < 0.001 versus bow milk)

**Fig. 2:**
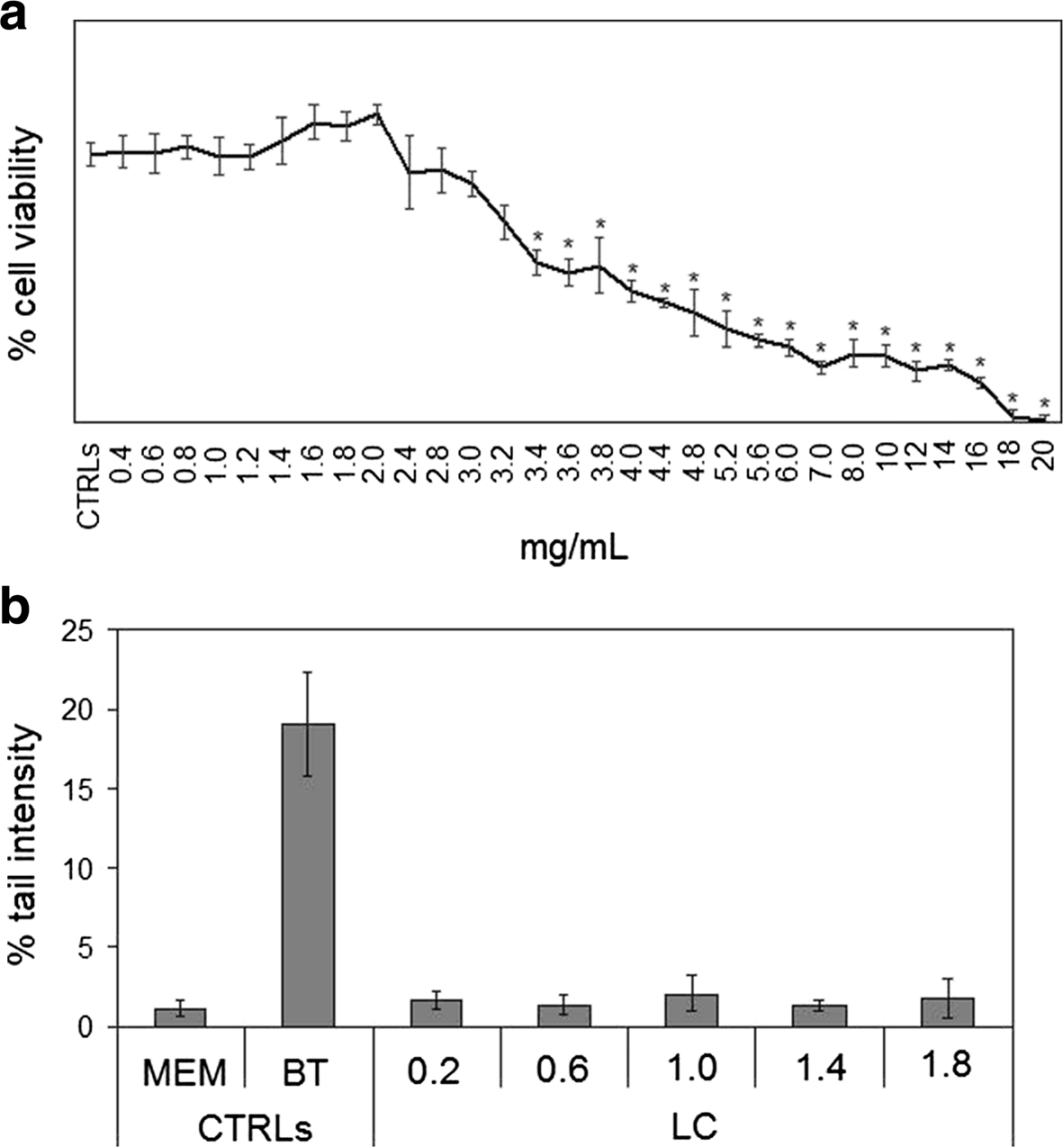
Effect of LC berry extract on HepG2 cells. **a** cell viability, measured by MTT assay; **b** genotoxic effects, valuated by Comet Assay. In **a** the values are reported as % viability of the control sample, and in **b** the values are reported as % tail intensity with cells in MEM as negative control and cells in 100 mM 1,2,4-benzentriol (BT) as positive control. Data are expressed as mean ± SD of three independent experiments performed in duplicate

The analysis of total phenolic content (TPC) and antioxidant capacity (ORAC) showed that the berry extract of LC have very high antioxidant properties. TCP value was 1574 ± 59 mgGAE/100gr dry weight (DW) and ORAC value was 18224 ± 1511 μmolTE/100gr DW (Fig. 1). In bow milk, know to have very low antioxidant properties and so used as negative control [18], TCP value was 17 ± 1 mgGAE/100gr and ORAC value was 855 ± 2 μmolTE/100gr DW. In LB berry extratcs, know to have very high antioxidant properties and so used as positive control [19], TCP value was 943 ± 231 mgGAE/100gr and ORAC value was 26502 ± 3807 μmolTE/100gr DW (Fig. 1). Increasing concentrations of extract were used to test the possible toxic effect in dependence on concentration. MTT assay showed that from 0.4 to 3.0 mg/mL extract concentration did not change cell viability of HepG2 after 24 h of culture (Fig. 2a). Higher concentrations reduced progressively cell viability, the IC50 value was 9.55 mg/mL. Even, 1.6, 1.8, and 2.0 mg/mL extract concentration increased slightly (about 8 ± 2 %) cell viability. Comet assay highlighted that after only 4 h, BT treatment induced genotoxic effect with 19.07 ± 0.69 % of tail intensity respect to untreated cells (MEM medium), used as negative control (Fig. 2b). No genotoxic effect was obtained with 0.2, 0.6, 1.0, 1.4, and 1.8 mg/mL LC extract concentrations (Fig. 2b). The results lead us to use 1.8 mg/mL LC berry extract for the subsequent experiments.

### *Lycium Chinense* berries target acid sphingomyelin and changes sphingomyelin species

**Fig. 3:**
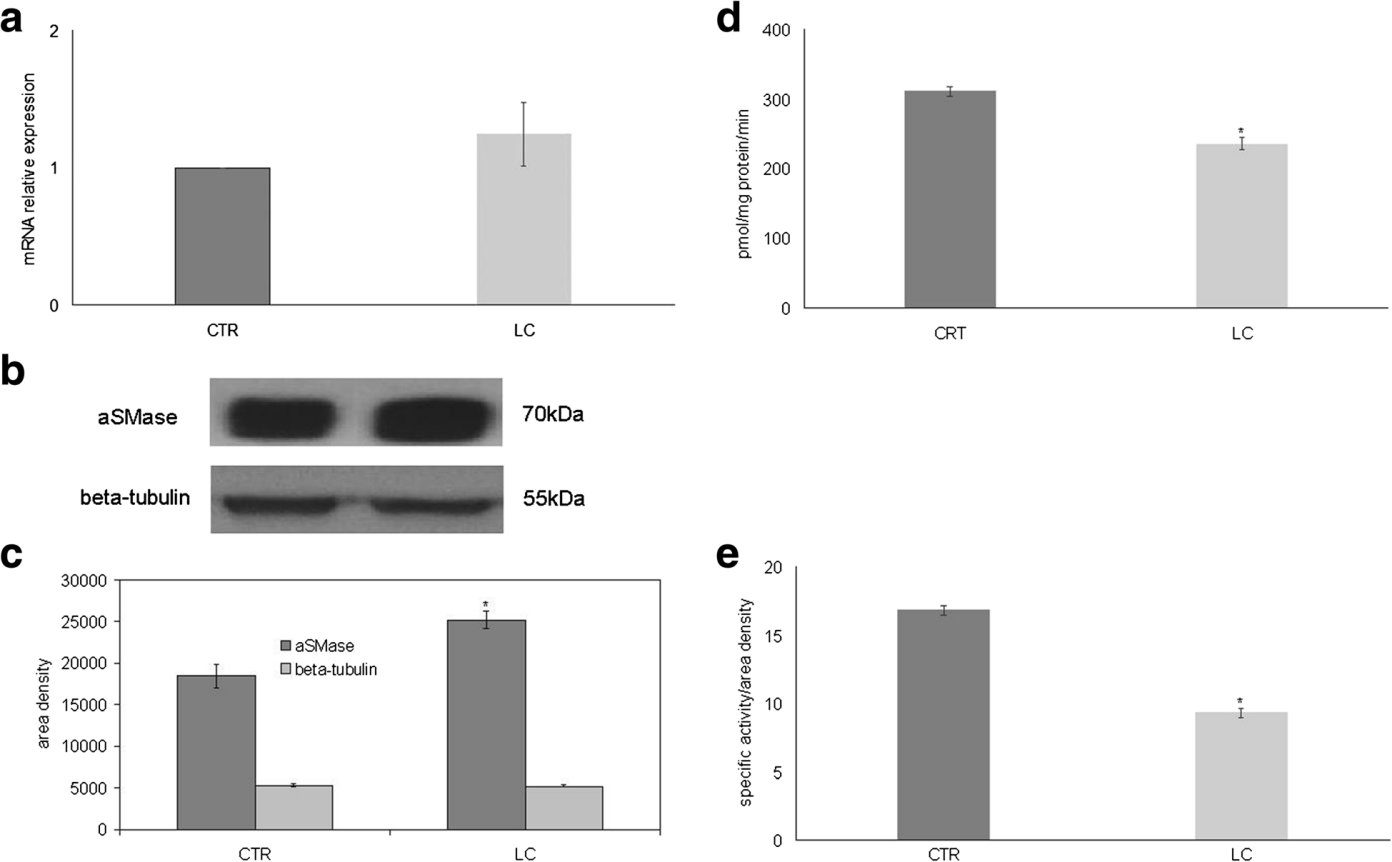
Effect of LC berry extract on acid sphingomyelinase. **a** gene expression evaluated by RT-PCR as fold of increase of mRNA expression relative to control sample. **b** immunoblotting; the position of the 70 kDa protein for aSMase and 48 kDa for beta-tubulin was indicated in relation to the position of molecular size standards. **c** area density evaluated by densitometry scanning and analyzed with Scion Image. **d** enzymatic activity; the data are expressed as pmol/mg protein/min. **e** enzymatic activity/ area density; the data are expressed as pmol/mg protein/min referred to area density of immunoblotting analyzed as reported in **c**. Data are expressed as area/mg protein. (Significance, *P < 0.001 versus control sample)

Treatment of the cells with LC berry extract did not change significantly aSMase gene expression (Fig. 3a). The ability of LC berries to influence aSMase protein content was tested next. Importantly, experiments of immunoblotting demonstrated that the LC extract increased the aSMase amount (Fig. 3b). The band density of aSMase, corresponding to 70 kDa apparent molecular weight, was 1.37 times higher in LC sample than in CRT sample (Fig. 3c). Therefore aSMase was sensitive to LC berries. Thus, we then tested aSMase activity and the results showed that LC berries were active in inhibiting the specific enzyme activity; the value was reduced 1.34 times in comparison with that of control sample (Fig. 3d). Furthermore, with regard the enzyme activity in relation to the band density of immunoblotting, it was evident that the aSMase activity decreased 1.81 times (Fig. 3e).

**Fig. 4:**
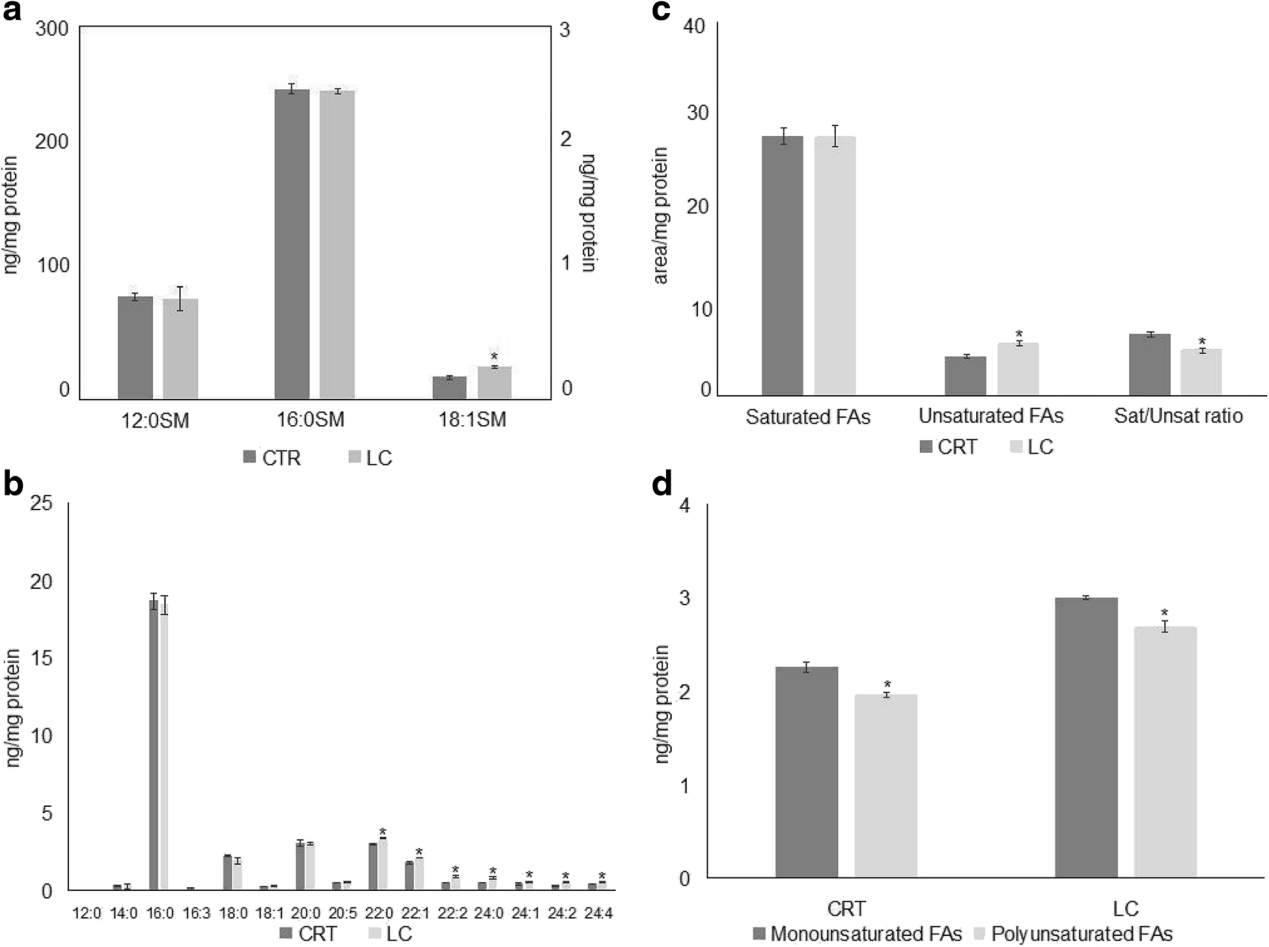
Effect of LC berry extract on sphingomyelin species. **a** sphingomyelin (SM) species studied by using 12:0 SM, 16:0 SM, and 18:1 SM external calibrators. Data are expressed as nmol/mg protein. Left ordinate, 16:0 SM, and 18:1 SM; right ordinate, 12:0 SM. **b** SM species studied by evaluating the areas of all the peaks identified on the basis of their molecular weight. Data are expressed as area/mg protein **c** total saturated and unsaturated fatty acids (FAs) and saturated/unsaturated ratio. Data are expressed as area/mg protein. **d** total monounsaturated and polyunsaturated FAs. Data are expressed as area/mg protein. Data are expressed as area/mg protein. (Significance, *P < 0.001 versus control sample)

Because we concluded of our experiments that LC berries inhibited aSMase activity, we investigated their effects on SM species to better understand the molecular mechanism regulated by LC berries. We analysed SM species in the cells treated with LC, by using 12:0 SM, 16:0 SM, and 18:1 SM external calibrators and the results were compared with those of untreated cells. The results show that the value of 12:0 SM and 16:0 SM did not change and 18:1 SM increased 3 % value (Fig. 4a). To have a deeper insight of SM species containing saturated or unsaturated fatty acids (FAs), we evaluated the areas of all the peaks identified on the basis of their molecular weight and we analyzed their values in relation to protein content. A total of twenty-two species were investigated: 12:0 SM, 14:0 SM, 16:0 SM, 16:1 SM, 16:3 SM, 18:0 SM, 18:1 SM, 18:2 SM, 20:0 SM, 20:1 SM, 20:2 SM, 20:5 SM, 22:0 SM, 22:1 SM, 22:2 SM, 22:3 SM, 24:0 SM, 24:1 SM, 24:2 SM, 24:3 SM, 24:4 SM, 26:0 SM. Fifteen peaks were detected (Fig. 4b). Significant differences in the levels of various lipid molecular species were found between CTR and LC samples. LC sample resulted richer in very long chain FAs (from 20:0 to 24:4) than CRT sample. Among the intermediate length acyl chains (18 and 20), only 18:1 was higher in LC sample in comparison CRT sample. Then we compared the changes in the total levels of SM species containing saturated and unsaturated FAs. As reported in Fig. 4c, the SM saturated FAs did not change and SM unsaturated FAs were 1.35 times higher in LC than in CRT sample. Thus, saturated/unsaturated FAs ratio was 6.60 + 0.28 in CRT sample and 4.89 ± 0.21 in LC sample (Fig. 4c). Among unsaturated FAs, the total monounsaturated FA SM species increased 1.33 times, the total polyunsaturated FA SM species increased 1.37 times.

### *Lycium Chinense* berries change gene expression

**Fig. 5:**
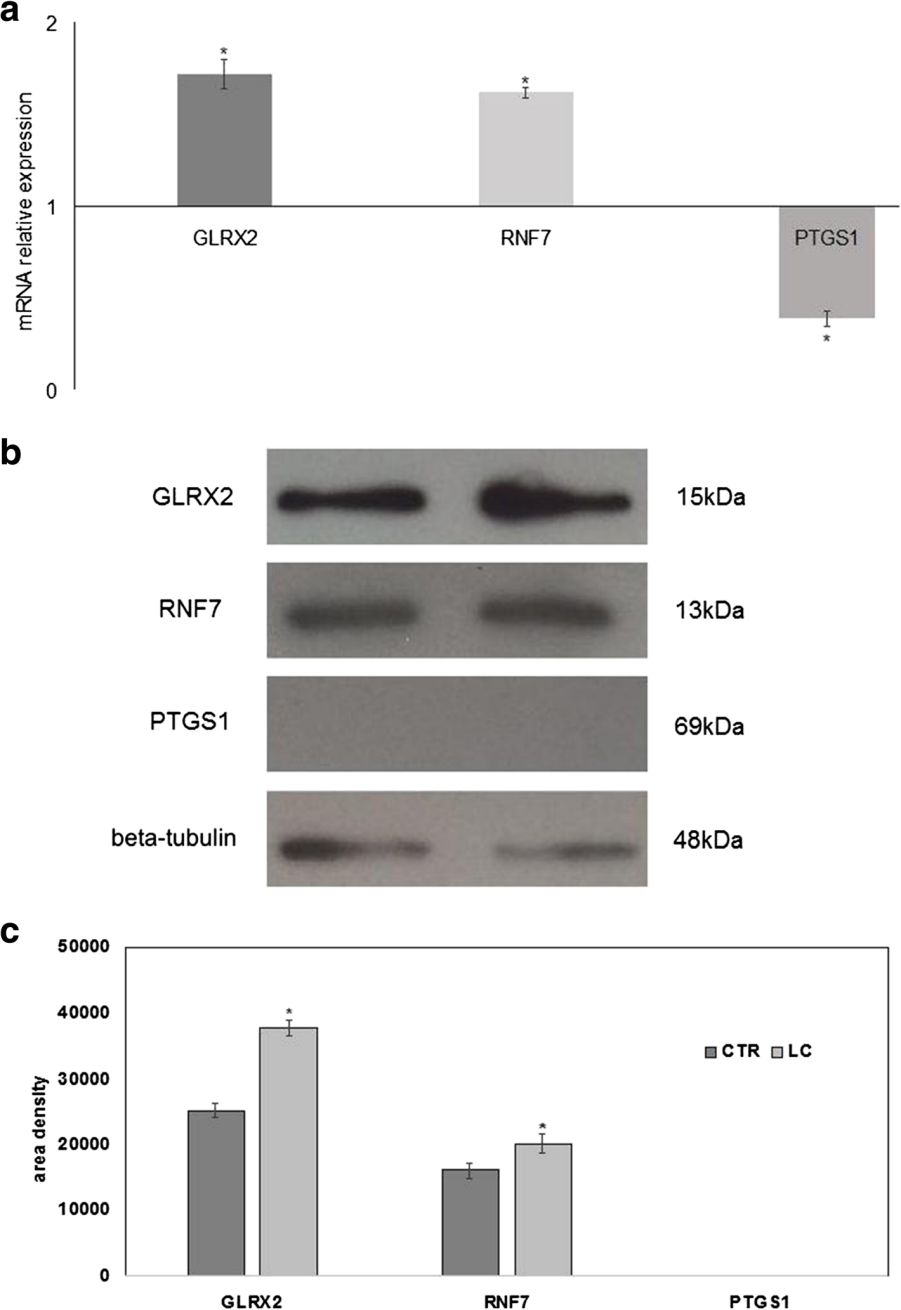
Effect of LC berry extract on gene and protein expression of HepG2 cells. The study was performed in control (without LC) and experimental cells (with LC) collected after 24 h of culture. **a** RTqPCR analysis of a panel of 96 genes involved in oxidative stress, proliferation, apoptosis and cancer was used. The results are normalized with the levels of the GAPDH and express as mRNA of experimental sample versus control sample. **b** immunoblotting analysis, the position of the 15 kDa protein for GLRX2, 13 kDa for RNF7, 69 kDa for PTGS1, and 48 kDa for beta-tubulin is indicated in relation to the position of molecular size standards. **c** area density is evaluated by densitometry scanning and analyzed with Scion Image. The results of GLRX2 and RNF7 are normalized with the levels of beta-tubulin. Data are expressed as the mean ± S.D. of 3 independent experiments performed in threeplicates. (Significance, *P < 0.001 versus control sample)

In order to define the specific contributions of LC berries to the HepG2 cell fate, we hypothesized that LC berries could play a role in the control of gene expression. Thus, to text the influence of LC berries on gene expression, we performed quantitative Real-Time PCR analysis with a panel of 96 genes involved in oxidative stress, proliferation, apoptosis and cancer. mRNA levels were normalized with respect to GAPDH, chosen as internal control. The results showed that LC berries changed only Glutaredoxin 2 (GLRX2), Ring Finger Protein 7 (RNF7), and Prostaglandin H synthase 1 (PTGS1). As shown in Fig. 5a, where the gene expression is referred to that of untreated cells, GLRX2 and RNF7 were over-expressed and PTGS1 was down-expressed. Following these finding, we investigated whether the proteins synthesized by these specific genes had the similar behavior. We found that GLRX2 and RNF7 increased 1.50 and 1.24 times, respectively. PTGS1 band protein was absent in control and LC samples (Fig. 5b, c).

## Discussion

Despite the ongoing evolution of the field of SM cycle-drug interaction there is a great need to investigate into the role of medicinal plants in SM metabolism, due to their increasing use. This led us to study aSMase as possible target of LC, traditionally used in Eastern countries and now spread all over the world. Cardiovascular, pulmonary, liver and neurological diseases together to environmental stress-induced diseases in which aSMase may be associated were widely described [20]. aSMase was described to have opposing roles in determining the response to drugs. Amitriptyline, generally known for its antidepressant properties, was used also for the treatment of various diseases thanks to its effect on aSMase inhibition [21]. Andrieu-Abadie et al. [22] demonstrated that L-carnitine blocked the doxorubicin-induced activation of the aSMase. The studies presented herein clearly established that LC berries inhibited aSMase activity in HepG2 cells. In the same time, LC berries changed the composition in SM species. In fact, the cells treated with LC berry extract presented a higher content of very long and unsaturated FAs in comparison with CRT sample. This is relevant for cell health, considering that the unsaturated FA level was inversely correlated with the risk of different pathologies [23, 24]. Our results suggested the possibility that aSMase inhibition LC berries-induced might be responsible for the reduction of unsaturated FA SM catabolism. How LC berries target aSMase is really hard to establish at the moment. It is possible that is occurred thanks to the antioxidant properties of LC berries. This is consistent with the observation that neutralization of cellular ROS using antioxidants markedly attenuated aSMase activation and ceramide-induced apoptosis [25]. On the other hand, the studies presented herein clearly demonstrated that LC berries regulated the expression of specific genes protecting from apoptosis. Dumitru et al. [26] showed that pharmacological inhibition of the aSMase abolished the apoptotic effect of gemcitabine in glioma cells. We revealed for the first time that in a panel of 96 genes involved in oxidative stress, proliferation, apoptosis and cancer only three genes were changed, GRX2, RNF7, and PTGS1. In particular GRX2, RNF7 genes were over-expressed and their correspondent protein content increased. GRX2 is an isozyme of glutaredoxin1 thioltransferase that acts as redox sensor and controls thiol/disulfide balance in cells [27]. Zhang et al. [28] demonstrated that GRX2 protected cells from apoptosis. RNF7, known as SAG (Sensitive to Apoptosis Gene), has non-enzymatic antioxidant activity [29] by protecting cells from apoptosis [30]. Thus LC could have an anti-apoptotic role in HepG2 cells. Notably PTGS1, known as cyclooxygenase-1(Cox.1), has been described to be involved in the progression of cancer [31]. Importantly, our results showed that despite the PTGS1 gene expression was found in the control sample, no protein expression was present in the HepG2 cells. Accordingly, Schmidt et al. reported that HepG2 cells did not express COX-1 protein, evaluated by immunoblotting [32]. It is possible to suppose that in hepatoma cancer cells the PTGS1 gene is normally expressed but there is an error in protein synthesis. Thus, LC down-expressed the PTGS1 gene but did not influence PTGS1 protein. These data would predict that LC could protect from apoptosis and not play an anti-cancer role in HepG2 cells.

## Conclusion

In conclusion, the results present in this study demonstrate that LC berries are useful for cell health by acting via aSMase and SM species modulation.

## Methods

### Cells and LC plants

The human hepatocellular carcinoma HepG2 cell line was purchased from Istituto Zooprofilattico Sperimentale della Lombardia e dell’Emilia Romagna ‘Bruno Ubertini’ (Brescia, Italy). LC plants were grown in Foligno, Umbria Region, Italy.

### Materials

Eagle’s Minimum Essential Medium (MEM), L-glutamine, trypsin, and ethylenediaminetetraacetic acid disodium and tetrasodium salt (EDTA) were from Microtech Srl (Pozzuoli, NA, Italy). Fetal Bovine Serum (FBS) and penicillin-streptomycin were from Thermo Fisher Scientific (Waltham, MA, USA). Antibiotics, sodium pyruvate, Dulbecco’s phosphate buffered saline pH 7.4 (PBS) were from Invitrogen Srl (Milan, Italy). Dimethyl sulfoxide (DMSO), ethanol, hydrochloric acid, sodium chloride, and sodium hydroxide were purchased from Carlo Erba Reagenti Srl (Milan, Italy). 6-hydroxy-2,5,7,8-tetramethylchroman- 2-carboxylic acid (Trolox), Trypan blue solution 0.4 %, 1,2,4-benzenetriol (BT), staurosporine, tris(hydroxymethyl)-aminomethane (Tris), Triton X100, valinomycyn, Folin Ciocalteu, 2,20-azobis (2-methylpropionamide) dihydrochloride (AAPH), 3-[4,5-dimethyl-2-thiazolyl]-2,5- diphenyl-2-tetrazolium bromide (MTT), methanol, 3-(4,5-dimethyl-thiazol-2-yl)-2,5-diphenyltetrazolium bromide, 2-propanol, metyl-tert-butyl ether, formic acid, chloroform were from Sigma-Aldrich Srl (St. Louis, MO, USA). Lipid standards 16:0 SM, 18:1 SM, and 24:0 SM were purchased from Avanti (Avanti Polar, Alabaster, AL). Anti-aSMase was from Abcam (Cambridge, UK). Anti-GLRX2, anti-RNF7, and anti-PTGS1 were from Termo Scientific (Rochford, USA). Horseradish peroxidase-conjugated goat anti-rabbit secondary antibodies was from Santa Cruz Biotecnology (California, USA).

### LC berry extract

LC berries were homogenized in physiological solution with Ultra Turrax T25 Basic homogenizer (Ika Labortechnick, Staufen, Germany) at room temperature for 1 min and centrifuged at 3150 g for 30 min. The supernatant was used for experiments.

### Total phenolic content (TPC)

The TPC of LC berry extracts was determined using the Folin–Ciocalteu colorimetric method described by Rashidinejad et al. [33] with modifications [34]. Commercial cow milk was used as negative control [18] and commercial LB berry extract [19] was used as positive control.

### Antioxidant Assay by Oxygen Radical Absorbance Capacity (ORAC)

The ORAC-fluorescein (ORAC-FL) assay was based on the procedure of Persichetti et al. [35] with slight modifications. The hydrophilic and lipophilic fractions were extracted. 2,20-azobis (2-methylpropionamide) dihydrochloride (AAPH) was used as a peroxyl radical generator, Trolox was used as a reference antioxidant standard, and fluorescein was used as a fluorescent probe. A 100 μL volume of diluted sample, blank or Trolox calibration solution (10–80 μmol) was mixed with 1 mL of fluorescein (80 nM); then, 200 μL of each mixture was placed in a well of the microplate. The microplate was placed in the reader and pre-incubated for 15 min at 37 °C. To each well, 60 μL of AAPH was automatically added to initiate the reaction. The fluorescence was measured every 60 sec. ORAC values in Trolox equivalents (TE [μmol/L g]) were calculated using the standard linear regression curve with Trolox concentration in the range 10–80 μM.

### Cell culture and treatments

HepG2 cells were grown in monolayer cultures in 25 cm^2^ tissue flasks, with MEM supplemented with 10 % heat-inactivated FBS, 1 mmol/L of sodium pyruvate, 2 mM of L-glutamine and antibiotics (100 U/ml penicillin, 100 μg/ml streptomycin). The cells were maintained in a cell incubator at 37 °C in a humidified atmosphere containing 5 % CO_2_. When the cells reached 80–90 % of confluence, the routine culture medium was aspirated and the HepG2 cells were washed with PBS 1X. The cells were then harvested by 0.05 % trypsin in 0.02 % Na_4_EDTA for 5 min at 37 °C, and suspended in 1:3 supplemented growth medium to be maintained in the exponential growth phase. For the experiments, the cells were counted by using trypan blue dye exclusion assay, seeded and cultured in absence or presence of LC berry extracts at different concentrations and for different times.

### Viability by MTT assay

HepG2 cells were seeded onto 96-well plate at a density of 1 × 10^4^ cells/well with MEM complete medium. After 24 h fresh complete medium was replaced for treatment with different concentrations (from 0.4 to 2 mg/mL) of LC berry extracts for 24 h. Cellular viability was measured using MTT assay according to Denizot and Lang [36]. Cell viability was expressed as a percentage relative to the control cells. The IC50 value was calculated by ED50plus Excel program.

### Comet assay

HepG2 cells were treated with different concentrations (0.2, 0.6, 1.0, 1.4, and 1.8 mg/mL) of LC berry extract for 4 h. Negative (MEM) and positive (100 mM 1,2,4-benzentriol) controls were included. The Comet assay was carried out basically following the original procedure [37], with minor modifications [38]. The comets in each microgel were analyzed (blind), at 200× magnification, with an epifluorescent microscope (BX41, Olympus Co., Tokyo, Japan) under a 100 W high-pressure mercury lamp (HSH-1030-L, Ushio Inc., Tokyo, Japan), using appropriate optical filters (excitation filter 510–550 nm and emission filter 590 nm). The microscope, equipped with a high sensitivity black and white CCD camera (PE2020, Pulnix Europe Ltd., Basingstoke, UK), was connected to a computerized analysis system (“Comet Assay III”, Perceptive Instruments, Suffolk, UK). The tail intensity was used to evaluate DNA damage. A total of 100 randomly selected comets (50 cells/replicate spot) were evaluated for each experimental point. For each independent test, the median tail intensity of 50 cells/spot was assessed and the average of 2 replicated spots was calculated as a summary statistic [39].

### Protein concentration and western Blotting

Protein concentration was determined according to the method of Bradford [40]. 40 μg HepG2 proteins were submitted to 10 % SDS (sodium dodecyl sulfate)-polycrylamide gel for SMase and PTGS1 detection, and to 12 % SDS for GLRX2 and RNF7 detection at 200 V for 60 min according to Codini et al. [41]. Briefly, proteins were transferred onto 0.45 μm cellulose nitrate strips membrane (Sartorius Stedim Biotech S.A.) in transfer buffer for 1 h at 100 V at 4 °C. Membranes were blocked with 5 % (*w/v*) non-fat dry milk in PBS, pH7.5 for 1 h at room temperature. The blot was incubated overnight at 4 °C with anti-aSMase antibody (1:1000). The blot was treated with horseradish peroxidase-conjugated goat anti-rabbit secondary antibodies (1:5000). SuperSignal West Pico Chemiluminescent Substrate (ThermoFisher Scientific) was used to detective chemiluminescent (ECL) HRP substrate. The apparent molecular weight of anti-aSMase was calculated according to the migration of molecular size standards. The area density of the bands was evaluated by densitometry scanning and analyzed with Scion Image.

### aSMasi activity assay

HepG2 cells were suspended in 0.1 % NP-40 detergent in PBS, sonicated for 30 s on ice at 20 watt, kept on ice for 30 min and centrifuged at 16,000 *g* for 10 min. The supernatants were used for aSMase assay. 60 μg/10 μl proteins were incubated with 10 μl HMU-PC substrate for 10 min at 37 °C. The reaction was stopped by adding 200 μl stop buffer. The fluorescence of 6-hexadecanoyl-4-methylumbelliferone (HMU) was measured with FLUOstar Optima fluorimeter (BMG Labtech, Germany), by using the filter set of 4-methylumbelliferone (MU), 360 nm excitation, and 460 nm emission. The fluorimeter was calibrated with MU in stop buffer.

### Lipid extraction

Lipid extraction was performed as reported by Lazzarini et al. [42] with modifications. The LC mother solution were suspended in Tris 10 mM, pH 7.4, and diluted with 1 ml methanol. Three milliliters ultrapure water and 3 ml MTBE were added. Each sample was vortexed for 1 min and centrifuged at 3000 × g for 5 min. The supernatant was recovered. The extraction with MTBE was repeated on the pellet and the supernatant was added to the first. The organic phase was dried under nitrogen flow and resuspended in 500 μl of methanol.

### Ultrafast liquid chromatography–tandem mass spectrometry

The 16:0 SM, 18:1 SM, 24:0 SM, standards were prepared as reported by Lazzarini et al. [42]. Standards were dissolved in chloroform/methanol (9:1 vol/vol) at 10 μg/mL final concentration. The stock solutions were stored at −20 °C. Working calibrators were prepared by diluting stock solutions with methanol to 500:0, 250:0, 100:0, and 50:0 ng/mL final concentrations. Twenty microliters of standards or lipids extracted from serum was injected after purification with specific nylon filters (0.2 μm). Analyses were carried out as reported by as reported by Lazzarini et al. [42] using the Ultra Performance Liquid Chromatography system tandem mass spectrometer (Applied Biosystems, Italy). The lipid species were separated, identified, and analyzed as previously reported [42, 43]. The samples were separated on a Phenomenex Kinetex phenyl-hexyl 100 A column (50 × 4.60-mm diameter, 2.6-μm particle diameter) with a precolumn security guard Phenomenex ULTRA phenyl-hexyl 4.6. For SM, column temperature was set at 50 °C and flow rate at 0.9 mL/min. Solvent A was 1 % formic acid; solvent B was 100 % isopropanol containing 0.1 % formic acid. The run was performed for 3 min in 50 % solvent B and then in a gradient to reach 100 % solvent B in 5 min. The system needed to be reconditioned for 5 min with 50 % solvent B before the next injection. The SM species were identified by using positive turbo-ion spray and modality multipole-reaction monitoring as reported by Lazzarini et al. [42, 43].

### PCR array analysis

HepG2 cells were used for total RNA extraction performed by using RNAqueous®-4PCR kit (Ambion Inc., Austin, Texas) as previously reported [44]. Samples were treated with RNase-free DNase to prevent amplification of genomic DNA possibly present. Samples were dissolved in RNAse–free water and total RNA amount was quantified by measuring the absorbance at 260 nm (A_260_). The purity of RNA was evaluated by using the A260/A280 ratio. A260/A230 ratio also was used as indicator of chemical contaminants in nucleic acids. The extracted RNA was immediately frozen and maintained at -80 °C. Before cDNA synthesis, the integrity of RNA was confirmed by denaturing electrophoresis in TAE 1.2 % agarose gel. cDNA was synthesized using 1 μg total RNA for all samples by High-Capacity cDNA Reverse Transcription kit (Applied Biosystems, Foster City, CA, USA) under the following conditions: 50 °C for 2 min, 95 °C for 10 min, 95 °C for 15 s and 60 °C for 1 min for 40 cycles. RTqPCR was performed using Master Mix TaqMan®Gene Expression and 7.500 RT-PCR instrument (Applied Biosystems), targeting genes in Taqman Array 96 - Well Plate P/N: 4414250, SM phosphodiesterase 1 (SMPD1, Hs03679347-g1), and glyceraldehyde-3-phosphate dehydrogenase (GAPDH, Hs99999905-m1) (Thermo Fisher Scientific, MA, USA).

### Statistical analysis

All experiments were repeated three times in duplicate. Data were reported as the mean ± SD. The significance of treatment was analyzed by using the t-Student (*P value was < 0.001).
